# Achieving asymmetry parameter-insensitive resonant modes through relative shift–induced quasi-bound states in the continuum

**DOI:** 10.1515/nanoph-2023-0673

**Published:** 2024-01-18

**Authors:** Tian Sang, Qing Mi, Chaoyu Yang, Xianghu Zhang, Yueke Wang, Yongze Ren, Ting Xu

**Affiliations:** Department of Photoelectric Information Science and Engineering, School of Science, Jiangnan University, Wuxi 214122, China; National Laboratory of Solid-State Microstructures, College of Engineering and Applied Sciences and Collaborative Innovation Center of Advanced Microstructures, Nanjing University, Nanjing 210093, China

**Keywords:** metasurface, bound states in the continuum, resonant mode

## Abstract

High-Q resonances in metasurfaces, stemming from symmetry-protected bound states in the continuum (BICs), have proven to be effective for achieving high-performance optical devices. However, the properties associated with symmetry-protected BICs are inherently limited, as even a slight variation in the asymmetry parameter leads to a noticeable shift in the resonance location. Herein, we introduce the concept of relative shift–induced quasi-BICs (QBICs) within dimerized silicon (Si) meta-lattices (DSMs), which can be excited when a nonzero relative shift occurs, a result of in-plane inversion symmetry breaking and Brillouin zone folding within the structure. These QBICs have resonance locations that remain insensitive to variations in asymmetrical parameters. Additionally, their Q-factors can be flexibly tuned, benefiting from the inverse square dependence on asymmetrical parameters. In experiment, six DSMs with different relative shifts are fabricated and the asymmetry parameter-insensitive resonant modes under two orthogonal polarization states are experimentally observed in the optical communication waveband. Our results offer unique opportunities for constructing high-Q resonators with enhanced performances, which can be applied in various optical fields.

## 1 Introduction

Bound states in the continuum (BICs) are radiationless localized states with infinite lifetime and perfect confinement of energy even though they lie in the radiation continuum [1], [2]. Although BICs were first introduced in quantum mechanics by Neumann and Wigner in 1929 [3], they are essentially the wave phenomena and only in the past decade has their rich physics been employed to the photonic systems [4], [5]. Optical BICs are associated with the vanishing couplings between the resonant modes and all radiation channels, which can be interpreted in several equivalent ways in photonic structures [6]. Because BICs have no emission channels, they are dark modes and their output power to the environment is theoretically zero. In application, the energy of BICs can be emitted through quasi-BICs (QBICs), which are coupled to radiation channels with high yet finite radiative quality-factor (Q-factor). In photonics, it is essential to achieve QBICs for enhanced light–mater interactions that could enable low-threshold [7], [8] and ultra-coherent [9], [10] lasers, optical filters [11], [12], highly efficient absorbers [13], [14], [15], and optical chirality enhancement [16], [17], [18].

For periodic optical lattice, one interesting type of QBICs relies on the symmetry properties of both discrete mode and coexisting radiative continua, and it is classified as symmetry-protected QBICs [19]. The symmetry-protected QBICs are transformed from the symmetry-protected BICs, whose coupling between the bound states and continuum band is completely decoupled due to the symmetry incompatibility. Generally, planar optical systems possessing 180° rotational symmetry about the *z* axis (C_2_) will have the symmetry-protected BICs, and the introduction of symmetry breaking of the structure will switch the symmetry-protected BICs to QBICs to generate radiation channel [20]. For example, by breaking the up-down or in-plane symmetry of the metasurfaces such as grating [21], [22], nanorings [23], [24], nanorods [25], [26], [27], nanodisks [28], [29], and cross-shaped resonators [30], symmetry-protected QBICs with tunable linewidths can be achieved at normal incidence. Despite the achievements in employing symmetry-broken metasurfaces to create high-Q resonances for the development of next-generation devices, current design paradigms for exciting symmetry-protected QBICs in planar configurations face limitations for two primary reasons. Firstly, structural asymmetry perturbations necessitate the addition or removal of meta-atom constituents, and even slight variations in the structural asymmetry parameter of the meta-lattices result in noticeable shifts in resonance wavelengths [[Bibr j_nanoph-2023-0673_ref_022] [Bibr j_nanoph-2023-0673_ref_023] [Bibr j_nanoph-2023-0673_ref_024] [Bibr j_nanoph-2023-0673_ref_025] [Bibr j_nanoph-2023-0673_ref_026] [Bibr j_nanoph-2023-0673_ref_027] [Bibr j_nanoph-2023-0673_ref_028] [Bibr j_nanoph-2023-0673_ref_029]. This renders them unsuitable for applications involving gain materials operating at specific wavelengths, such as BIC lasers and fluorescence enhancement. In essence, precise control of the meta-atom asymmetry parameters is imperative for achieving resonance at the intended wavelength, which, in turn, escalates fabrication complexities. Secondly, while breaking the in-plane symmetry of the meta-atom unit cell can yield dual-band high-Q resonances, both resonances are excited simultaneously and cannot be selectively addressed [23], [28], [30]. Consequently, the pursuit of high-Q resonances characterized by insensitivity to structural asymmetry parameter variations in the meta-lattices, along with the capability to selectively excite resonant wavelengths, remains a pressing aspiration in the domain of flat-optics devices.

Herein, we propose the relative shift–induced QBICs to achieve asymmetry parameter insensitive high-Q resonant modes based on the dimerized silicon (Si) meta-lattices (DSMs). The QBICs can be excited as the relative shift of the Si meta-dimers is nonzero due to in-plane inversion symmetry breaking as well as the Brillouin zone folding of the structure. Crucially, the positions of these QBICs induced by relative shifts remain nearly unchanged due to their bonding mode characteristics. This unique property enables flexible tuning of their Q-factors even when the structural asymmetry parameter of the DSMs undergoes substantial alteration. Furthermore, these relative shift-induced QBICs can be selectively excited for two orthogonal polarization states, corresponding to the out-of-plane toroidal dipole (TD) and the in-plane TD modes for the *x* and *y* polarizations, respectively. Finally, our experimental validation confirms that these two wavelength-insensitive resonance modes can be selectively excited under two orthogonal polarization states by inducing relative shifts in the Si meta-dimers.

## 2 Results

### 2.1 Design principle for the proposed DSMs

**Figure 1:**
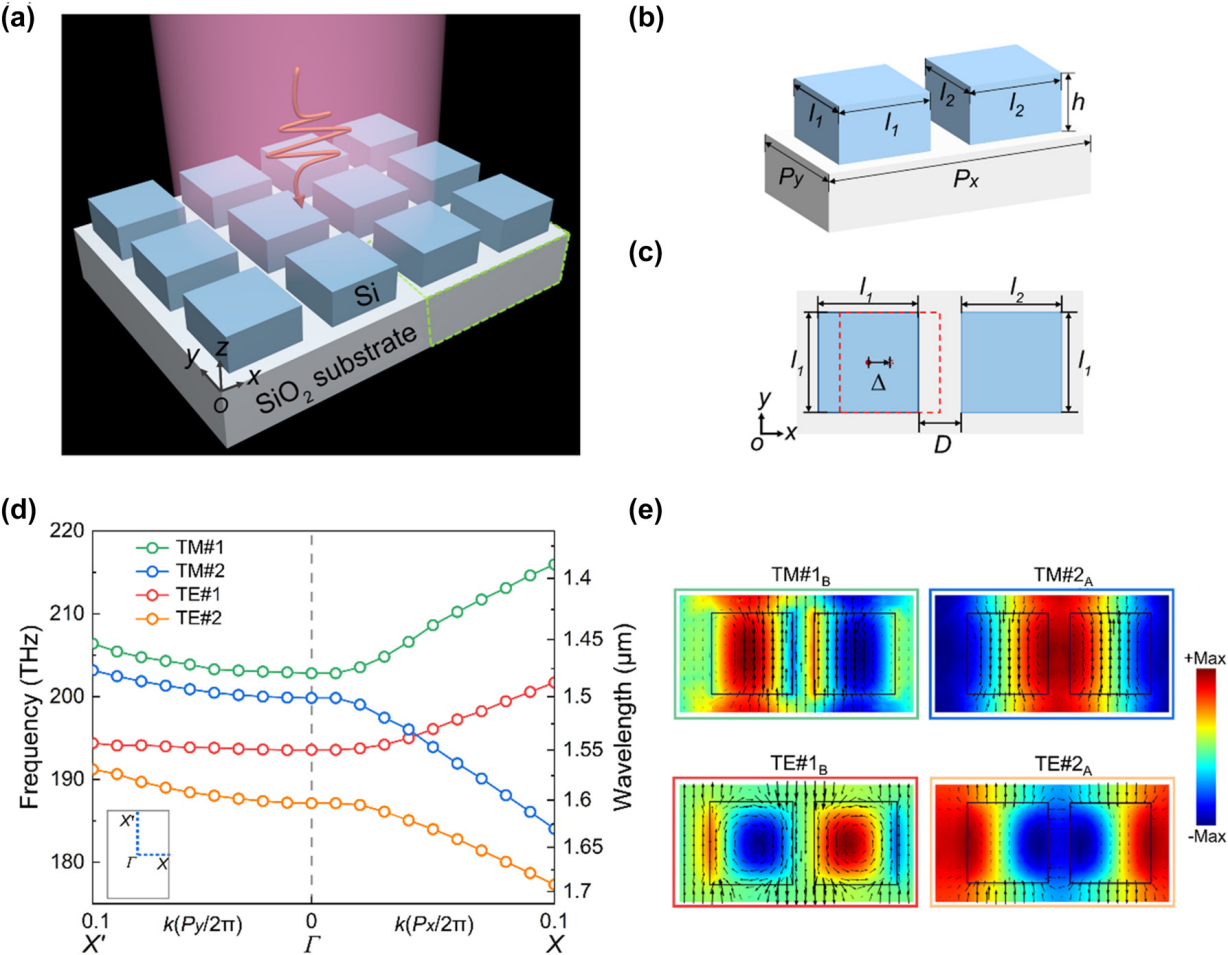
Schematic diagram of the proposed DSMs and its band properties. (a) Schematic diagram of the DSMs under the plane wave illumination, the supercell of the DSMs is indicated by the green dash line. (b) Schematic diagram of the supercell of the DSMs. (c) Vertical view of the supercell of the DSMs in the *xy* plane, where the shift of a Si disk from its center is indicated as Δ. (d) Band diagram of the DSMs along *X*′ − *Γ* − *X*. The parameters are *P*_*x*_ = 2*P*_*y*_ = 1000 nm, *l*_1_ = *l*_2_ = 350 nm, *h* = 220 nm, and *δ* = Δ/*D* = 0.34. (e) Electromagnetic field distributions of the DSMs at Γ point for the TM-like and TE-like modes. TM#1_B_ and TM#2_A_ are *z* component of electric-field *E*_*z*_ overlaid with the in-plane magnetic-field vector *H*_*xy*_; TE#1_B_ and TE#2_A_ are *z* component of magnetic-field *H*_*z*_ overlaid with the in-plane electric-field vector *E*_*xy*_.

Figure 1 shows the schematic diagram of the proposed DSMs and its band properties. As shown in Figure 1(b) and (c), the supercell of the DSMs consists of two Si disks supported by silica (SiO_2_) substrate. The Si meta-dimers in the supercell have the same height *h*, and their widths are *l*_1_ and *l*_2_, respectively. The periods of the supercell of the DSMs in the *x* and *y* directions are *P*_*x*_ and *P*_*y*_, respectively. The refractive indexes of Si and SiO_2_ are 3.48 and 1.47, respectively. As the two Si disks are located in the center of half area of the supercell, the distance between them along the *x* direction is *D*. The shift of a Si disk from its center along the and *x* direction is marked as Δ, and the structural asymmetry parameter of the DSMs can be described by the relative shift, which is defined as *δ* = Δ/*D*. In the case of *l*_1_ = *l*_2_ and *δ* = 0, the DSMs are degenerated into the simple lattice structure where its unit cell consists of a single Si disk, and there is no band structure in the wavelength region of interest for the structural parameters indicated in Figure 1. However, by introducing the relative shift with *δ* ≠ 0, the supercell is formed and the band structure of the DSMs can be realizable due to the in-plane inversion (C_2_) symmetry breaking as well as the Brillouin zone folding along the *x* direction [31], [32], [33]. Therefore, it is possible to excite the previously inaccessible modes (dark modes) by free-space illumination through the relative shift of the supercell of the DSMs.

Figure 1(d) shows the band structure of the DSMs with *δ* = 0.34 along the *X*′ − Γ − *X*. The dispersion curve of the DSMs shows TM-like mode (with strong *E*_*z*_ component and negligible *E*_*x*_, *E*_*y*_, and *H*_*z*_ components) and TE-like mode (with strong *H*_*z*_ component and negligible *H*_*x*_, *H*_*y*_, and *E*_*z*_ components). These TM-like and TE-like modes shows bonding (field highly confined in Si disks) and antibonding (field mainly trapped by air gaps) features, which are indicated as the subscript of *B* and *A*, respectively. That is, TM#1_B_ (TE#1_B_) indicates the TM (TE) bonding modes, and TM#2_A_ (TE#2_A_) indicates the TM (TE) antibonding modes. Figure 1(e) shows electromagnetic field distributions of the DSMs at Γ point for the TM-like and TE-like modes. As can be seen in Figure 1(e), the odd–even symmetry of the eigenmodes of the DSMs are different. The TM#1_B_ (TE#1_B_) modes have odd-like transverse electric-field (magnetic-filed) profiles, while the TM#2_A_ (TE#2_A_) modes have even-like transverse electric-field (magnetic-filed) profiles. Basically, these two sets of modes with the opposite parity are related to the electromagnetic duality, which reflects the symmetry of Maxwell’s equations with respect to the electric and magnetic components of electromagnetic waves [5].

**Figure 2:**
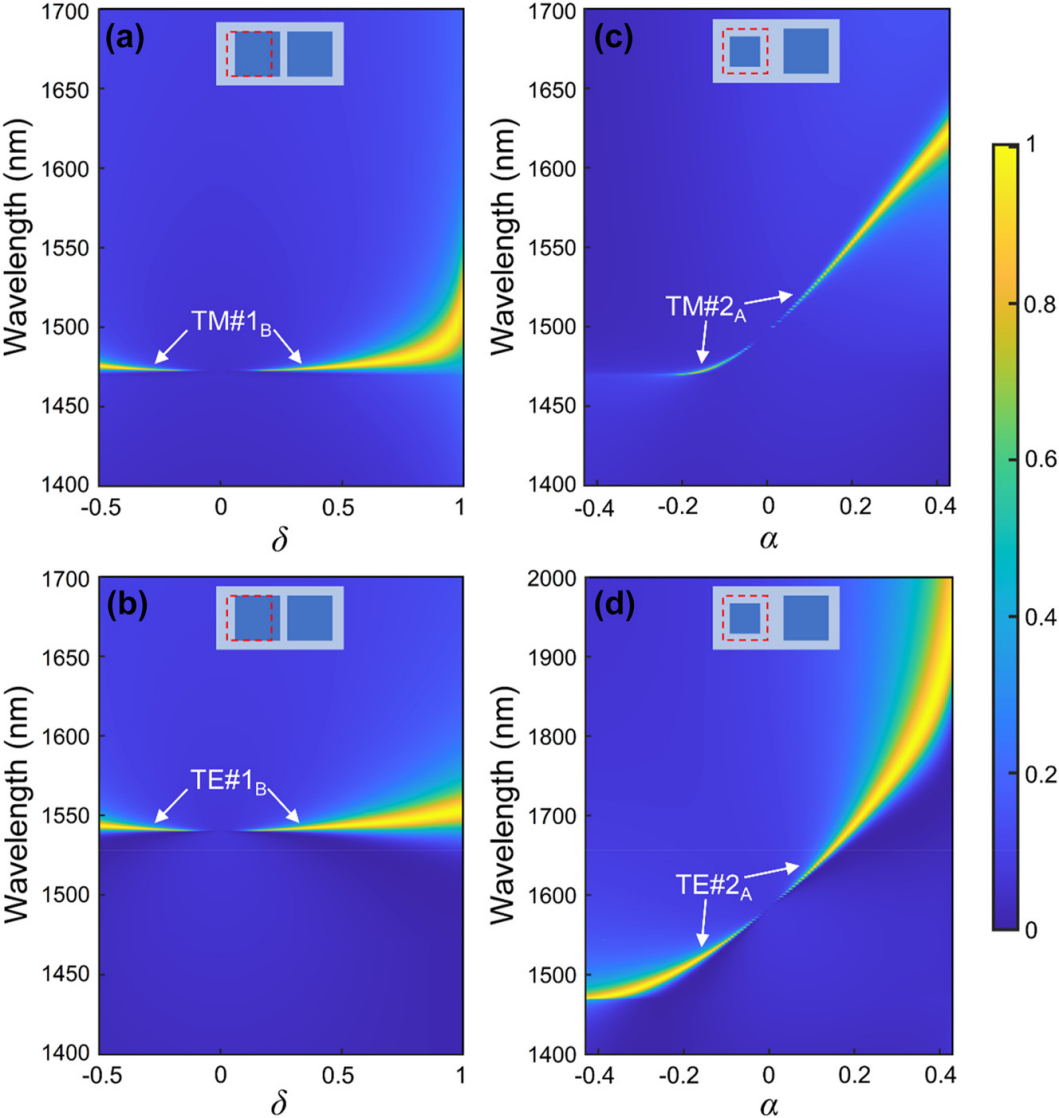
Reflection 2D maps of the DSMs as functions of the asymmetry parameters under normally incident plane waves, the schematic diagrams of the supercell of the DSMs are inserted in the figures; other parameters are the same as Figure 1. (a) and (b) are reflection 2D maps of the DSMs with *l*_1_ = *l*_2_ as functions of *δ* for the *x* and *y* polarizations, respectively. (c) and (d) are reflection 2D maps of the DSMs with *δ* = 0 as functions of *α* for the *x* and *y* polarizations, respectively, where *α* = (*l*_1_ − *l*_2_)/*l*_2_.

Figure 2 depicts two-dimensional (2D) reflection maps of the DSMs as functions of structural asymmetry parameters, considering normally incident plane wave illumination. All other parameters remain consistent with those presented in Figure 1. We evaluate two distinct categories of asymmetry parameters: the conventional parameter *α*, linked to in-plane inversion symmetry breaking in DSMs, and the relatively less-explored parameter *δ*, associated with mirror symmetry breaking within DSMs. Upon close examination of Figure 2, it becomes evident that for both *δ* = 0 and *α* = 0, no resonances manifest in either scenario, owing to the inherent simplicity of the lattice structure in DSMs. However, these initially nonresonant dark modes undergo a transformation into high-Q radiative modes as *α* or *δ* deviate from zero due to structural symmetry perturbation. It is noteworthy that the linewidths of these high-Q modes expand with increasing absolute values of *α* or *δ*. Of particular interest, when *δ* ≠ 0 and *α* ≠ 0 for DSMs, high-Q modes associated with both mirror symmetry breaking and in-plane inversion symmetry breaking can be excited simultaneously (see Supplementary S1, Supporting Information).

Specifically, Figure 2(a) and (b) shows reflection 2D maps of the DSMs with *l*_1_ = *l*_2_ as functions of *δ* for two orthogonal polarization states. In these figures, it can be seen that two QBICs modes can be excited with *δ* ≠ 0, which are associated with the eigenmodes of TM#1_B_ and TE#1_B_ for the *x* and *y* polarizations, respectively. For the *x* polarization, the relative shift of the DSMs leads to the in-plane inversion symmetry breaking of the magnetic field about the *y*–*z* plane. Conversely, for the *y* polarization, it corresponds to the in-plane inversion symmetry breaking of the electric field about the *y*–*z* plane. Consequently, two high-Q modes linked to two symmetry-protected BICs can be selectively switched under orthogonal polarization states. Figure 2(c) and (d) further illustrate that two QBICs modes can also be excited as the C_2_ symmetry is disrupted with *α* ≠ 0. These modes are associated with the eigenmodes of TM#2_A_ and TE#2_A_ for the *x* and *y* polarizations, respectively. It is noteworthy that in the case of *x* polarization in Figure 2(c), the high-Q mode of the DSMs tends to vanish as *α* falls below −0.3. This behavior arises from the fact that the (±1, 0) diffraction orders propagate in the substrate near the cutoff wavelength of 1470 nm, corresponding to the Rayleigh anomaly [34]. As *α* varies, it redistributes the field energy of the antibonding mode confined in the air gap, resulting in significant shift of the location of the high-Q modes. In contrast, the location of the high-Q modes is robust to the variation of *δ* due to their bonding mode characteristics, where field energy is well confined in the Si meta-dimers thus insensitive to the variation of *δ*. This property proves advantageous for the robust excitation of high-Q modes at specific wavelengths. In forthcoming studies, our focus will center on the optical performance of DSMs, particularly with regard to the less-discussed asymmetry parameter of relative shift *δ*.

### 2.2 Analysis on the excitation of relative shift-induced QBICs

**Figure 3:**
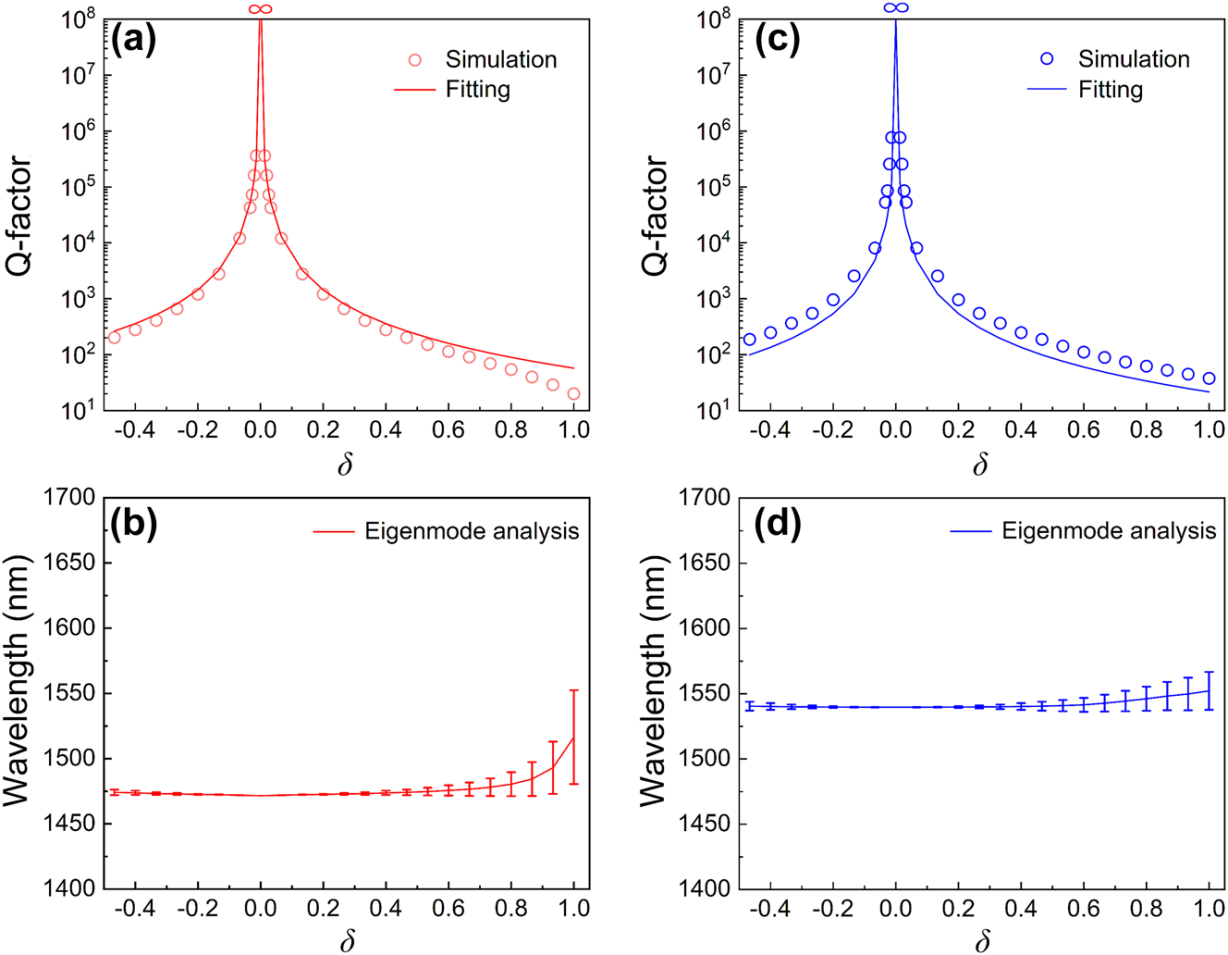
Optical performances of the DSMs as functions of the relative shift *δ. *(a) and (b) are Q-factor of the DSMs as function of *δ* for the *x* and *y* polarizations, respectively. (c) and (d) are the eigenmode responses of the DSMs as function of *δ* for the *x* and *y* polarizations, respectively; the error bars indicate the magnitude of the mode inverse radiation lifetime. Other parameters are the same as Figure 1.

Figure 3 shows optical performances of the DSMs as functions of the relative shift *δ*, with the remaining parameters remaining consistent with those presented in Figure 1. In Figure 3(a)–(c), the Q-factor of the reflection response of the DSMs are defined as *λ*_*r*_/∆*λ*, where *λ*_*r*_ is the resonant wavelength and ∆*λ* is its linewidth. According to the perturbation theory [20], the radiative quality factor *Q*_rad_ of a metasurface is inversely proportional to the inverse radiation lifetime *γ*_rad_, and it shows an inverse square dependence on the structural asymmetry parameter as long as the QBIC frequency is below the diffraction limit of the substrate. Therefore, the Q-factor of the DSMs related to the relative shift *δ* can be fitted as:

(1) $$Q_{\mathrm{fit}}=\eta \cdot \frac{cS}{\omega \cdot \delta^{2}},$$

where *η* is a proportionality constant, *c* is the speed of light in vacuum, *S* is the area of the supercell, and *ω* is the angular frequency. As illustrated in Figure 3(a)–(c), divergent radiative Q-factor that tends to infinity is occurred at *δ* = 0 for both the *x* and *y* polarizations, indicting the existence of the BICs. However, even a minimal relative shift leads to a significant reduction in the Q-factor of the DSMs. The fitting results for the Q-factor align closely with those obtained from simulations, for both *x* and *y* polarizations. Specifically, for small values of *δ*, the behavior of the DSMs’ Q-factor exhibits a clear inverse quadratic trend. Slight discrepancies emerge at larger *δ*, primarily due to deviations from the slight perturbation approximation.

Figure 3(b)–(d) shows the eigenmode responses of the DSMs as function of relative shift *δ* for the *x* and *y* polarizations, respectively. The complex eigenfrequency of the eigenmode containing real and imagery parts can be written as $\tilde{\omega }$ = Re( $\tilde{\omega }$ )+Im( $\tilde{\omega }$ ), where Re( $\tilde{\omega }$ ) corresponds the resonance location, and 2·Im( $\tilde{\omega }$ ) corresponds the mode inverse radiation lifetime. As can be seen in Figure 3(b)–(d), the resonance locations of the eigenmodes are insensitive to the variations of the relative shift *δ*, while the radiation leakage related to the mode inverse radiation lifetime can be gradually enhanced with the increase of |*δ|*, which are in accord with the reflection features of the DSMs shown in Figure 2(a) and (b). Therefore, controllable radiation leakage operating at the desired wavelength is feasible through the relative shift of the Si meta-dimers.

**Figure 4:**
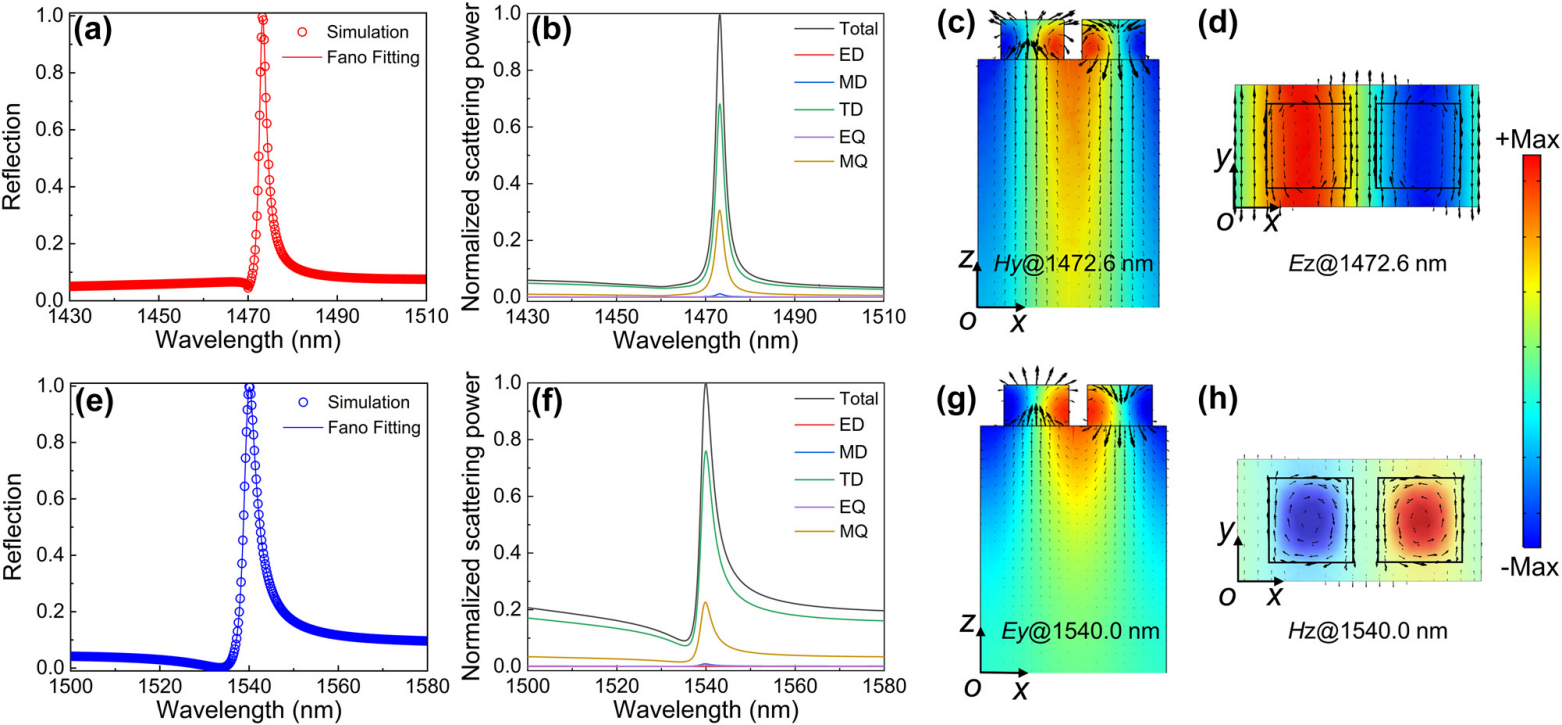
Optical properties of the DSMs with *δ* = 0.34; other parameters are the same as Figure 1. (a) and (e) are reflection responses of the DSMs for the *x* and *y* polarizations, respectively. (b) and (f) are the far-field scattering power for the *x* and *y* polarizations, respectively. (c) and (d) are near-field distribution at reflection peak of 1472.6 nm for the *x* polarization. (g) and (h) are near-field distribution at reflection peak of 1540.0 nm for the *y* polarization. Specifically, colors in (c) and (d) indicate the distributions of *y* component of magnetic-field *H*_*y*_ and *z* component of electric-field *E*_*z*_, respectively; black arrows in (c) and (d) indicate displacement current vector and magnetic-field vector, respectively. Colors in (g) and (h) indicate the distributions of *y* component of electric-field *E*_*y*_ and *z* component of magnetic-field *H*_*z*_, respectively; black arrows in (g) and (h) indicate magnetic-field vector and displacement current vector, respectively.

Figure 4 shows optical properties of the DSMs with *δ* = 0.34. All other parameters remain consistent with those presented in Figure 1. As the relative shift of the Si meta-dimers is associated with the interference between the discrete resonant mode and the broadband incident waves, the relative shift–induced QBICs can be described by the typical Fano formula [35]:

(2) $$R\left(\omega \right)=R_{0}+A_{0}\frac{{\left[q+2\left(\omega -\omega_{0}\right)/\kappa \right]}^{2}}{1+{\left[2\left(\omega -\omega_{0}\right)/\kappa \right]}^{2}},$$

where *ω*_0_ is the resonant frequency, *R*_*0*_ is the reflection offset, *κ* is the resonance linewidth, *A*_*0*_ is coupling constant between the discrete and continuum states, and *q* is the Breit–Wigner–Fano parameter determining asymmetry of the resonance profile.

Figure 4(a)–(e) shows reflection responses of the DSMs for the *x* and *y* polarizations, respectively. As can be seen in Figure 4(a)–(e), the fitting results of the analytic Fano formula are in good agreement with the reflection responses for both the *x* and *y* polarizations, validating the excitation of the QBIC-induced Fano resonance accompanied with the relative shift of the Si meta-dimers. To further understand the resonant mechanism of the relative shift–induced QBICs, the far-field scatterings and the near-field distributions of the DSMs for both the *x* and *y* polarizations are investigated. In calculation, the induced current density in the supercell is extracted to perform the multipole decomposition in Cartesian coordinate [36], [37], and the far-field scatterings of the DSMs are decomposed into five major components: electric dipole (ED), magnetic dipole (MD), toroidal dipole (TD), electric quadrupole (EQ), and and magnetic quadrupole (MQ) (see Supplementary S2, Supporting Information).

As shown in Figure 4(b)–(f), for both the *x* and *y* polarizations, it can be seen that the relative shift–induced QBICs can be regarded as the TD mode as the TD resonance provides the major contributions to the far-field scattering power among the multipoles. For the *x* polarization at reflection peak of 1472.6 nm, as shown in Figure 4(c) and (d), the resonance feature is obvious as the magnetic field and electric field are highly trapped by the supercell of the DSMs. In particular, as shown in Figure 4(c), the magnetic field is strongly enhanced and the displacement current forms two reversed loops between the center and the edge of the Si meta-dimers at the *x*–*z* plane, indicating the out-of-plane TD mode along the *z* axis [38]; the distribution of the magnetic-field vector with reverse direction along the *y* axis in Figure 4(d) also reveals the feature of the out-of-plane TD mode. For the *y* polarization at reflection peak of 1540.0 nm, as shown in Figure 4(g) and (h), the field pattern also exhibits the resonance properties due to its highly localized distribution. Specifically, as shown in Figure 4(g), the magnetic field vector’s distribution forms a clockwise loop in the center of the supercell at the *x*–*z* plane, signifying the presence of the in-plane TD mode along the *y*-axis. The reversed loops of the displacement current vector at the *x*–*y* plane in Figure 4(h) also align with the features of the in-plane TD mode. The congruence between the far-field scatterings and the near-field distributions of the DSMs confirms that the out-of-plane and in-plane TD modes are responsible for the excitations of the relative shift–induced QBICs for the *x* and *y* polarizations, respectively.

### 2.3 Experimental observations of asymmetry parameter-insensitive resonant modes

**Figure 5:**
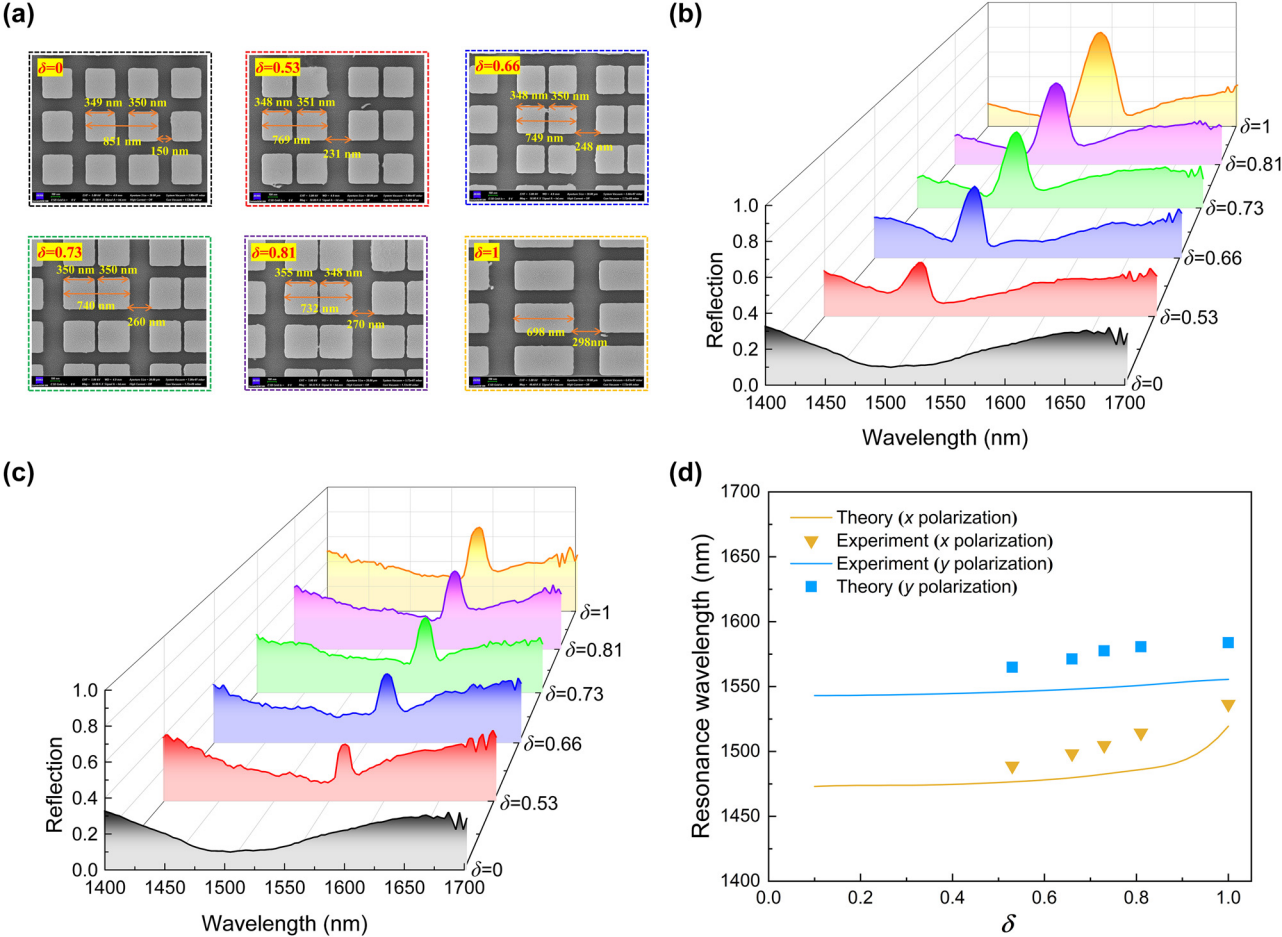
Experimental results of the DSMs. (a) SEM images of the fabricated DSMs with different relative shifts. (b) and (c) are the measured reflection responses of the DSMs for the *x* and *y* polarizations, respectively. (d) Simulated and measured resonance wavelengths of the DSMs as functions of the relative shifts of the Si meta-dimers.

Subsequently, we conduct experiments to validate the existence of relative shift–induced QBICs within the DSMs, where the resonance locations remain unaffected by variations in structural asymmetry parameters. In the experiment, the DSMs are fabricated by resist spin-coating, electron beam lithography (EBL), and inductively coupled plasma (ICP) etching techniques (see Supplementary S3, Supporting Information). Six types of samples with different *δ* are fabricated on a silicon-on-insulator (SOI) wafer. It’s worth noting that DSMs with 0 < *δ* < 0.5 are not fabricated because their resonance linewidths are too narrow to be detected in our measurement system. Scanning electron microscopy (SEM) images of the fabricated samples, displayed in Figure 5(a), reveal relatively well-matched structural parameters in alignment with the design. However, it’s important to note that, in comparison to DSMs supported by SiO_2_ substrates, the resonance locations of the SOI-based structures remain insensitive to variations in *δ*, but their linewidths are broadened and their Q-factors significantly reduced due to mode leakage into the Si substrate via open diffraction channels (see Supplementary S4, Supporting Information).

Figure 5(b) presents the measured reflection responses for the *x* polarization. As observed, at *δ* = 0, there exists only a low-reflection background. However, a relative shift of the Si meta-dimers induces a resonance above this background in the short-wavelength region. The linewidth of this resonance broadens with increasing *δ*, yet its location remains robust against variations in *δ*. It’s worth noting that the peak reflection is reduced, and the resonance linewidths are broadened in the measured responses, which can be attributed to material losses, surface roughness, and finite lateral dimensions of the samples [39]. In Figure 5(c), we can observe a relative shift-induced QBIC in the measured responses in the longer wavelength region, whose location remains insensitive to *δ* and can also have its linewidth tuned by adjusting the asymmetry parameters. Figure 5(d) demonstrates that the measured resonance wavelengths of the DSMs exhibit a tendency in line with the simulation results, albeit with a slight redshift in the resonance location. These deviations may arise from minor variations in parameters, such as refractive indices of materials or imperfections in the surface profile of the Si meta-dimers.

## 3 Conclusions

In summary, we have demonstrated relative shift–induced QBICs whose resonance locations are insensitive to the variation of the asymmetrical parameter by using the DSMs. The relative shift of Si meta-dimers will significantly alter the resonance linewidth due to the inverse square dependence on asymmetrical parameter, but the resonance location can be kept almost the same even for the large relative shift. The relative shift of Si meta-dimers results in the in-plane inversion symmetry breaking as well as the Brillouin zone folding of the structure, and two high-Q resonances can be selectively excited under the illuminations of two orthogonal polarization states. Crucially, these two high-Q resonances remain insensitive to variations in the relative shift of Si meta-dimers, thanks to their bonding mode characteristics with field energy well confined in the Si disks. These findings open up unique opportunities for the development of high-Q resonators with enhanced performance characteristics, with potential applications in nanolasers, optical switching, multichannel filtering, and other advanced optical devices.

## Supplementary Material

Supplementary Material Details
