# Supplementary material for: Achieving asymmetry parameter-insensitive resonant modes through relative shift–induced quasi-bound states in the continuum
Source: Nanophotonics. 2024 Jan 18;13(8):1369–77. doi: 10.1515/nanoph-2023-0673 (PMC11636510; doi:10.1515/nanoph-2023-0673)
Supplement: Supplementary file 1 — Supplementary Material Details [file j_nanoph-2023-0673_suppl_001.docx]

Supporting Information

Achieving asymmetry parameter-insensitive resonant modes through relative shift-induced quasi-bound states in the continuum

**Tian Sang**^1*^**,****Qing Mi**^1^**, Chaoyu Yang**^1^**, Xianghu Zhang**^1^**, Yueke Wang**^1^**, Yongze Ren**^2^**, Ting Xu**^2*^

*^1^Department of Photoelectric Information Science and Engineering, School of Science, Jiangnan University, Wuxi 214122, China*

*^2^National Laboratory of Solid-State Microstructures, College of Engineering and Applied Sciences and Collaborative Innovation Center of Advanced Microstructures, Nanjing University, Nanjing 210093, China*

*^*^Email: sangt@jiangnan.edu.cn,* [*xuting@nju.edu.cn*](mailto:xuting@nju.edu.cn)

**Supplementary S1: Reflection responses of the DSMs with δ≠0 and α≠0**

**
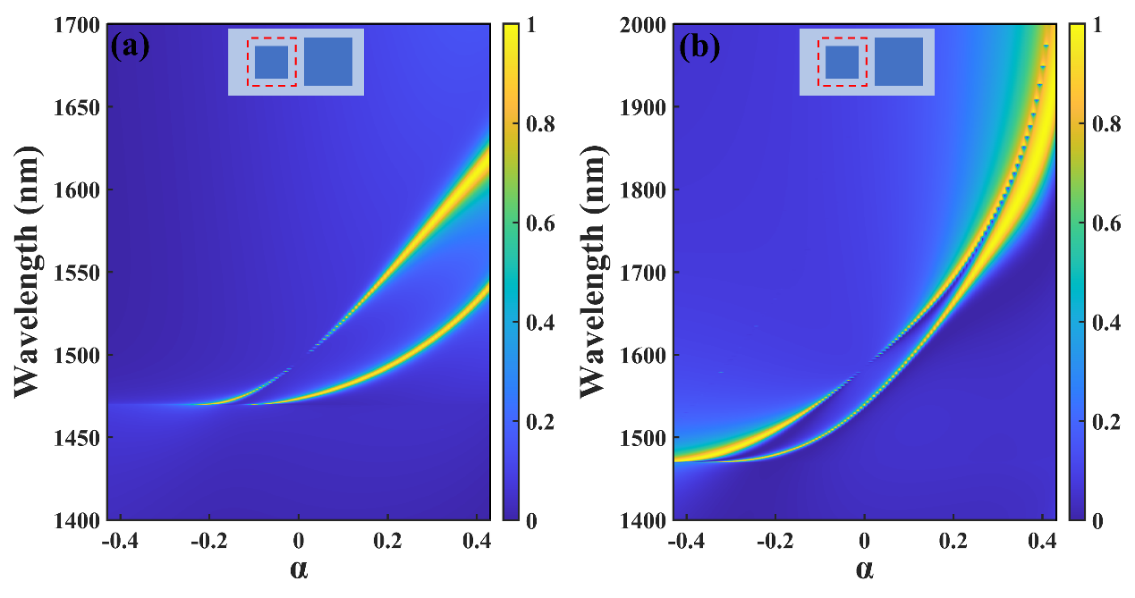
**

**Fig. S1.** Reflection 2D maps of the DSMs with *δ*=0.34 as functions of the asymmetry parameter *α*, other parameters are the same as Fig. 1. (a) and (b) corresponds to the *x* and *y* polarizations, respectively.

Figure S1 shows reflection responses of the DSMs with *δ*=0.34 as functions of the asymmetry parameter *α*. Other parameters are: *P_x_*=2*P_y_*=1000 nm, *h*=220 nm. As can be seen in Fig. S1, dual resonant modes associated with mirror symmetry breaking and in-plane inversion symmetry breaking can be excited simultaneously for two orthogonal polarization states. However, only the relative shift-induced QBICs can be excited at *α*=0 for both the *x* and *y* polarizations.

**Supplementary S2: Multipole decomposition of the DSMs**

In the Cartesian coordinate system, the multipole moments for the far-field scattering can be calculated according to the displacement current density ***j*** in the supercell of the DSMs [1]:

$P=\frac{1}{i\omega}\int jd^{3}r$ (1)

$M=\frac{1}{2c}\int(r\times j)d^{3}r$ (2)

$T=\frac{1}{10c}\int[(r\cdot j)r-2r^{2}j]d^{3}r$ (3)

$Q_{\alpha,\beta}^{(e)}=\frac{1}{i2\omega}\int[r_{\alpha}j_{\beta}+r_{\beta}j_{\alpha}-\frac{2}{3}(r\cdot j)\delta_{\alpha,\beta}]d^{3}r$ (4)

$Q_{\alpha,\beta}^{(m)}=\frac{1}{3c}\int[(r\times j)_{\alpha}r_{\beta}+(r\times j)_{\beta}r_{\alpha}]d^{3}r$ (5)

where ***P***, ***M***, ***T***, $Q_{\alpha,\beta}^{(e)}$, and $Q_{\alpha,\beta}^{(m)}$ are the moments of ED, MD, TD, EQ and MQ, respectively; *c* is the speed of light in vacuum, and *α*,*β* = *x*,*y*,*z*. Here the charge density *ρ*, which usually appears in the definition of ED and MQ, has been replaced with displacement current density ***j*** via charge-conservation relationship of $i\omega\rho+\nabla\cdot j=0$. The scattering power of the induced multipole moments contributing to the far-field response can be written as:

$I=\frac{2\omega^{4}}{3c^{3}}\left|P\right|^{2}+\frac{2\omega^{4}}{3c^{3}}\left|M\right|^{2}+\frac{2\omega^{6}}{3c^{5}}\left|T\right|^{2}+\frac{\omega^{6}}{5c^{5}}\sum_{\alpha,\beta} \left|Q_{\alpha,\beta}^{(e)}\right|^{2}+\frac{\omega^{6}}{40c^{5}}\sum_{\alpha,\beta} \left|Q_{\alpha,\beta}^{(m)}\right|^{2}+o(\omega)$ (6)

where the first, second, third, fourth, and fifth terms correspond to the scatterings of ED, MD, TD, EQ, and MQ, respectively; the last term is the higher-order scattering term that can be generally ignored. By using Eqs. (1)-(6), the normalized far-field scatterings of the DSMs from different multipoles can be obtained.

**Supplementary S3: Fabrication procedures of the DSMs samples**


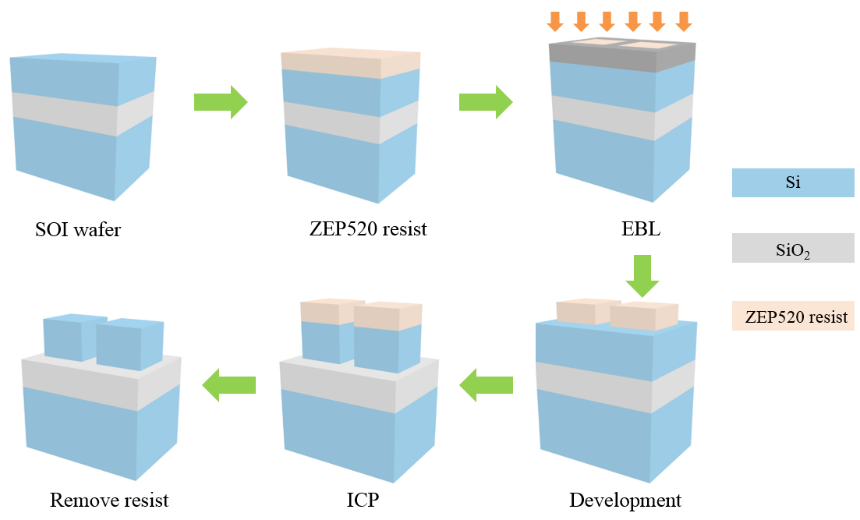


**Fig. S2.** Fabrication procedures of the DSMs based on the SOI wafer.

**Supplementary S4: Resonant properties of the DSMs based on SOI wafer**

Figure S3 shows reflection responses of the SOI-based DSMS, where the thickness of the buried SiO_2_ layer is 2 μm, and the thickness of Si substrate is 725 μm. As can be seen in Fig. S3(a), comparing with the SiO_2_-based DSMs shown in Fig. 4(a) with *δ*=0.34, the peak reflection of the SOI-based DSMS is low with peak value of 24.5% instead of 100%, this is due to the suppression of the resonance lifetime associated with the diffraction channels opening in Si substrate [2,3]. In addition, comparing with the SiO_2_-based DSMs, the linewidths of the SOI-based DSMS are larger and their Q factors are lower because more light energy will penetrate and leak into the Si substrate due to its high refractive index. However, similar as the SiO_2_-based DSMs, the resonance locations of the SOI-based DSMS are only slightly shifted even if the relative shift *δ* are significantly altered. In the case of the SOI-based DSMs for the *y* polarization, as shown in Fig. S3(b), the reflection responses exhibit the similar trends as that of the *x* polarization. Note there is a reflection dip around 1421 nm due to the high-order resonances associated with the (-1,0) and (1,0) transmission diffraction.


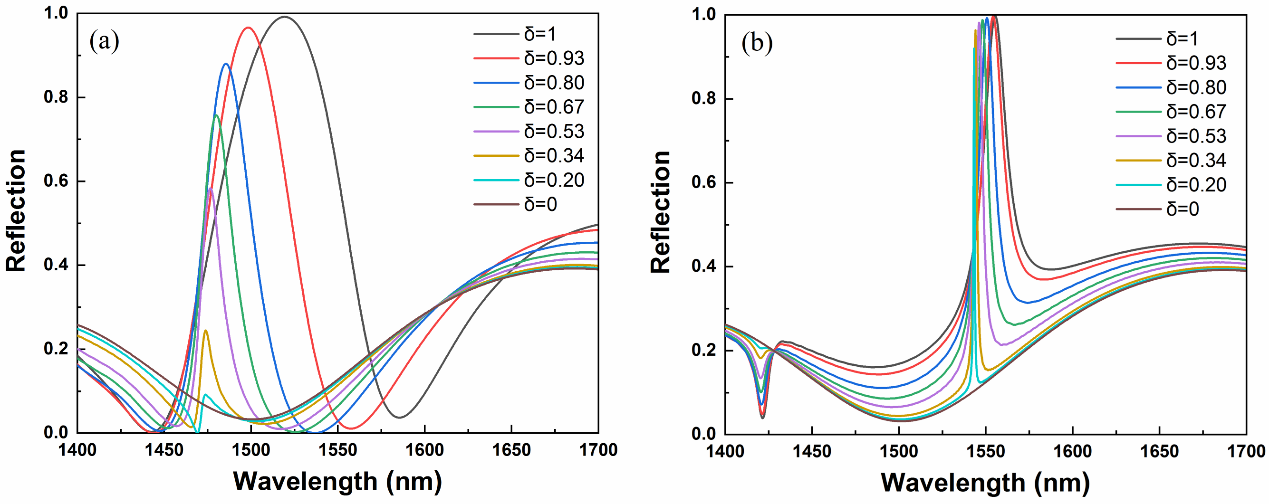


**Fig. S3.** (a) and (b) are reflection responses as functions of relative shift of the SOI-based DSMs for the *x* and *y* polarizations, respectively. The parameters are: *P_x_*=2*P_y_*=1000 nm, *l_1_*=*l_2_*=350 nm, and *h*=220 nm.

**References**

1. T. Kaelberer, V. A. Fedotov, N. Papasimakis, D. P. Tsai, and N. I. Zheludev, “Toroidal dipolar response in a metamaterial,” Science **330**(6010), 1510-1512 (2010).

2. T. Sang, G. Chen, Y. Wang, B. Wang, W. Jiang, T. Zhao, and S. Cai, “Tunable optical reflectance using a monolithic encapsulated grating,” Opt. Laser Technol. **83**, 163-167 (2016).

3. Z. F. Sadrieva, I. S. Sinev, K. L. Koshelev, A. Samusev, I. V. Iorsh, O. Takayama, R. Malureanu, A. A. Bogdanov, and A. V. Lavrinenko, “Transition from optical bound states in the continuum to leaky resonances: role of substrate and roughness,” ACS Photonics **4**(4), 723-727 (2017).
